# Comprehensive genomic evaluation of advanced and recurrent breast cancer patients for tailored precision treatments

**DOI:** 10.1186/s12885-023-11442-9

**Published:** 2024-01-16

**Authors:** Mirai Ido, Kimihito Fujii, Hideyuki Mishima, Akihito Kubo, Masayuki Saito, Hirona Banno, Yukie Ito, Manami Goto, Takahito Ando, Yukako Mouri, Junko Kousaka, Tsuneo Imai, Shogo Nakano

**Affiliations:** 1 Department of Surgery, Division of Breast and Endocrine Surgery, Aichi Medical University Hospital, 1-1 Yazakokarimata, Nagakute city, 480-1195 Japan; 2 Cancer Center, Aichi Medical University Hospital, Nagakute city, Japan

**Keywords:** Breast cancer, Comprehensive genomic profiling, Next generation sequencing and FoundationOne CDx

## Abstract

**Aim:**

The aim of this study was to investigate genetic alterations within breast cancer in the setting of recurrent or *de novo* stage IV disease.

**Patients and methods:**

: This study included 22 patients with recurrent breast cancer (n = 19) and inoperable *de novo* stage IV breast cancer (n = 3). For next generation sequencing, FoundationOneCDx (F1CDx) (Foundation Medicine Inc., Cambridge, MA, USA) was performed in 21 patients and FoundationOneLiquid CDx was performed in 1 patient.

**Results:**

Median age was 62.9 years (range, 33.4–82.1). Pathological diagnoses of specimens included invasive ductal carcinoma (n = 19), invasive lobular carcinoma (n = 2), and invasive micropapillary carcinoma (n = 1). F1CDx detected a median of 4.5 variants (range, 1–11). The most commonly altered gene were *PIK3CA* (n = 9), followed by *TP53* (n = 7), *MYC* (n = 4), *PTEN* (n = 3), and *CDH1* (n = 3). For hormone receptor-positive patients with *PIK3CA* mutations, hormonal treatment plus a phosphoinositide 3-kinase inhibitor was recommended as the treatment of choice. Patients in the hormone receptor-negative and no human epidermal growth factor receptor 2 expression group had significantly higher tumor mutational burden than patients in the hormone receptor-positive group. A *BRCA2* reversion mutation was revealed by F1CDx in a patient with a deleterious germline *BRCA2* mutation during poly ADP ribose polymerase inhibitor treatment.

**Conclusion:**

Guidance on tailored precision therapy with consideration of genomic mutations was possible for some patients with information provided by F1CDx. Clinicians should consider using F1CDx at turning points in the course of the disease.

## Introduction

Modern advanced diagnostic technology that includes radiological and genomic approaches enables patients to receive personalized precision care. For breast cancer, tailored treatment strategies consider not only clinicopathological findings but also genomic profiling results. It is estimated that 5 to 10% of women breast cancer cases are linked to germline mutations [1]. The well-known germline mutation associated with high breast cancer risk includes *BRCA1/2* mutation. For the recurrent breast cancer patients harboring those germline mutations, the treatment of olaparib, a poly (ADP-ribose) polymerase inhibitor, is considered as an effective targeted therapy [2]. And alpelisib, phosphoinositide 3-kinase inhibitor, shows efficacy in *PIK3CA*-mutated recurrent breast cancer patients [3]. Unfortunately, for breast cancer patients who carry other mutations such as *PTEN, TP53, CDH1 and MYC*, no effective medication is introduced. Under the circumstances, 10 types of genome screening systems are currently available [4]. And only 3 of them are approved by the national health insurance system: OncoGuide™ NCC Oncopanel System (Sysmex Corporation, Kobe, Japan) [5], FoundationOne®CDx (F1CDx), and FoundationOne® Liquid CDx (F1CDx-Liquid) (Foundation Medicine Inc., Cambridge, MA, USA) [6–8]. Although the OncoGuide™ NCC Oncopanel System can evaluate germline mutations in blood samples, it can evaluate fewer variants that F1CDx (114 versus 324 variants, respectively). F1CDx is approved as a companion diagnosis method.

In this study, we performed comprehensive genomic profiling using F1CDx and F1CDx-Liquid for 22 patients with breast cancer, which included 19 patients with recurrent disease and 3 patients with *de novo* stage IV disease. This study was conducted using real-world clinical data. There are few publications about this type of investigation in the field of breast cancer treatment. The clinically applicable data presented in this study might contribute to improving treatment strategies for advanced and metastatic breast cancer.

## Patients and methods

This study was conducted to compile the comprehensive genomic profiling data using F1CDx and F1CDx-Liquid retrospectively and to elucidate the specific genomic alterations of the breast cancer patients. And we weighted those genomic date against the conventional intrinsic subtype of breast cancer.

### Patients

A total of 22 patients with breast cancer who had a F1CDx examination date between January 2020 and May 2022 were enrolled in this study. Among these 22 patients, 17 patients underwent surgery at Aichi Medical University Hospital (Nagakute, Aichi, Japan), 1 patient at Marumo Hospital (Nagoya, Aichi, Japan), and 1 patient at Aichi Cancer Center Hospital (Nagoya, Aichi, Japan). They were referred to our institution when recurrence was diagnosed. The remaining 3 patients were diagnosed with *de novo* stage IV cancer on the first visit to the outpatient clinic of our hospital. Patients’ past medical history and family history of cancer were summarized in Table 1. Information on TNM stage [9], operative procedure, and lines of previous chemotherapy were collected from medical records.

**Table 1:** Characteristics of the study patients

	Number of patients
Age at F1CDx* (years)	62.9 (33.4–82.1)
**Past medical history**	
Metachronal breast cancer	0
Ovarian cancer	0
Adult T-cell leukemia	1
**Family medical history**	
Breast cancer	3
Ovarian cancer	0
Other cancers^†^	3
**TNM stage at initial diagnosis** ^**‡**^	
I	2
II	10
III	6
IV	3
Unknown	1
**Surgical procedure**	
Mastectomy	15
Partial resection	4
None^§^	3
**Lines of previous chemotherapy** ^**||**^	
0	9
1	2
2	11
**Tissue source**	
Biopsy specimen^¶^	16
Surgery specimen	6

* Median (range)

^†^Other cancers included lung cancer (n = 1), cervical cancer (n = 1), and endometrial cancer (n = 1)

^‡^ UICCs, 8th edition

^§^ Patients with clinical stage IV disease at diagnosis

^||^Previous chemotherapy includes the number of chemotherapeutic treatments that had affected the tumor biology before taking the samples that were served for F1CDx and F1CDx-Liquid

^¶^Biopsy specimen include skin (n = 4), lymph node (n = 3), lung (n = 2), liver (n = 1), remnant breast (n = 1) and primary breast tumor (n = 2)

### Tumor pathology

Pathological assessment of specimens from all patients was performed by the Department of Pathology at Aichi Medical University Hospital. Histologic type was determined according to World Health Organization criteria [10]. Estrogen receptor (ER) or progesterone receptor (PgR) positivity was defined as moderate-to-intense nuclear staining of ≥ 10%. Human epidermal growth factor 2 (HER2) positivity was defined based on fluorescence in situ hybridization (FISH) with the PathVysion®HER-2 DNA Probe kit (Abbott Pharmaceutical Co. Ltd., Lake Bluff, IL, USA); results were assessed according to the manufacturer’s instructions. For FISH score assessment, a HER2:centromeric probe 17 (CEP 17) ratio of ≥ 2 was defined as positive for HER2 amplification. A programmed death-ligand 1 (PD-L1) immunohistochemistry (IHC) assay was performed for patients whose hormonal and HER2 status was ER(-), PgR(-), and HER2(-). In this subgroup, patients who are PD-L1–positive might benefit from treatment with a immune checkpoint inhibitor such as pembrolizumab or atezolizumub [11]. For the SP142 IHC assay for PD-L1 (VENTANA OptiView PD-L1 (SP142), F. Hoffmann-La Roche Ltd., Basel, Switzerland), the percentage of immune cells was recorded as the percentage of tumor area (consisting of tumor cells and associated intratumoral and contiguous peritumoral stroma) occupied by immune cells with discernible PD-L1 staining of any intensity. Positivity was defined as ≥ 1% immunoreactive cells [12]. For the 22C3 IHC assay for PD-L1 (Dako, Carpinteria, CA, USA), the combined positive score (CPS) was defined as the number of PD-L1–stained cells (which includes tumor cells, lymphocytes, and macrophages) divided by the total number of viable tumor cells and multiplied by 100. Positivity was defined as CPS ≥ 1 [13].

### BRCA1/2 germline mutations

For the assessment of germline mutations *BRCA1/2*, BRACAnalysis CDx® (Myriad Genetics, Inc., Salt Lake City, UT, USA) was used in all patients. This commercialized test was first established for ovarian cancer [14]. This examination was conducted at the time of diagnosis of recurrence for 19 patients and at time of diagnosis of *de novo* stage IV cancer for 3 patients. The results of this examination were confirmed by attending physicians.

### FoundationOne^®^CDx

Comprehensive genomic analyses were performed using F1CDx in all but 1 patient for whom F1CDx-Liquid was used [6–8]. Each attending physician decided on whether to use F1CDx or F1CDx-Liquid. The specimens in the present series subjected to F1CDx analysis had (mean ± standard deviation) 38.6 ± 11.2% tumor nuclei. The F1CDx-targeted next-generation sequencing (NGS) platform has been previously described and validated. The methodology was demonstrated by Frampton et al. [7]. After formalin-fixed, paraffin-embedded samples were retrieved from biopsy or surgical specimens, 10 unstained sections and 1 hematoxylin and eosin stained section with thickness of 5 μm and tumor area > 25 mm^2^ were delivered to Foundation Medicine Inc. All samples were confirmed as adenocarcinoma and contained a minimum of 20% tumor cells. They were graded as Pass or Qualified. F1CDx applies NGS across the entire coding DNA of 324 genes proven to be solid tumor drivers. In addition, both tumor mutational burden (TMB) and microsatellite instability (MSI) was evaluated. TMB was presented as the number of mutations per megabase (Mut/Mb) of sequenced DNA. MSI was classified as stable, intermediate, or high. If the DNA sequence could not be determined with confidence, the result for TMB and MSI was reported as “cannot be determined.”

### FoundationOne^®^ Liquid CDx

F1CDx-Liquid is an NGS-based in vitro diagnostic method targeting 324 genes that is approved by the U.S. Food and Drug Administration. It uses circulating cell-free DNA (cfDNA) isolated from plasma derived from anti-coagulated peripheral whole blood of patients with cancer. All coding exons of 309 genes are targeted; select intronic or non-coding regions are targeted in 21 of these genes. Additionally, select intronic or non-coding regions are targeted in 15 genes, resulting in 324 total targeted genes. The assay detects substitutions, indels, genomic rearrangements, copy number alterations (CNAs) including amplifications and losses, and genomic signatures including blood TMB and MSI [8].

### Statistical analysis

BellCurve for Excel (Social Survey Research Information, Tokyo, Japan) was used for statistical analyses. The Mann-Whitney U test was performed for line of chemotherapy, number of pathogenic variants, variants of unknown significance, and tumor mutational burden score. The significance level was defined as 0.05. To assess the correlation between line of chemotherapy and number of variants, linear regression was used. Correlations were expressed with Pearson correlation coefficients. The correlation coefficient was scaled with range from − 1 to + 1, where 0 indicates no linear association [15].

## Results

Table 1 demonstrates the characteristics of the patients enrolled in this study. Median age at the date of F1CDx and F1CDx-Liquid examination was 62.9 years (range, 33.4–82.1 years). All patients were female. One patient had a history of adult T-cell leukemia, which had been completely cured before breast cancer treatment. Three patients had a family history of breast cancer and 1 patient each had a family history of lung cancer, cervical cancer, and endometrial cancer. Three patients were diagnosed with *de novo* stage IV breast cancer; they received hormonal or chemotherapeutic treatment without surgical intervention.

Among 22 patients enrolled, 2 patients experienced receptor conversion at sites of metastases (Table 2).

**Table 2:** Pathological findings

	Number of patients
**Pathological type**	
Invasive ductal carcinoma	19
Invasive lobular carcinoma	2
Invasive micropapillary carcinoma	1
Hormonal and HER2 status*	
HR(+), HER2(-)	13
HR(-), HER2(-)	7
HR(+), HER2(+) → HR(+), HER2(-)	1
HR(+), HER2(-) → HR(-), HER2(-)	1
**PD-L1 status** ^†^	
22C3 Positive	2
Negative	1
SP142 Positive	2
Negative	3

* ER(-) plus PgR(-) was described as HR(-). ER(+) plus PgR(+) was described as HR(+)

^†^PD-L1 immunohistochemistry was performed for the 7 patients whose hormonal and HER2 status was HR(-) and HER2(-)

For those 2 patients, the F1CDx assay was carried out using the specimen biopsied at the sites of metastases, metastatic lymph node, and lung. Tumor cells at those sites had demonstrated receptor conversion. In the patient who had lung metastasis, HER2 overexpression in the primary breast cancer shifted to HER2-negative status. The fluorescence in situ hybridization (FISH) score, which indicates the HER2 status of this patient, changed from 2.64 (primary lesion) to 1.1 (metastatic lesion).

For all patients, the BRACAnalysis CDx^®^ genetic test was used to detect germline *BRCA1/2* mutations [14]. A germline *BRCA2* mutation was found in only 1 patient who presented with a *BRCA2* reversion mutation during treatment with olaparib, a poly(ADP-ribose) polymerase inhibitor.

Three patients (13.6%) had a high TMB score (> 10 mut/Mb). None of them received immunotherapy although patients with a high TMB would benefit from T-cell checkpoint inhibitors (Table 3). It is reported that hypermutation occurs in about 5% of all breast cancers [16].

**Table 3:** Additional information

	Number of patients
***BRCA1/2*** **mutation**	
Yes	1*
No	21
**MSI**	
High	0
Stable	20
CD	2
**TMB**	
≥ 10	3
0–9	17
CD	2
**TMB score (muts/Mb)**	
Median (range)	3.0 (0–11)

CD: Data cannot be determined due to the low quality of the sample

* *BRCA2* reversion mutation was recognized in this patient

Variant alterations are summarized in Table 4. All patients had at least 1 identified variant that was either verified or likely pathogenic, with a median of 4.5 variants identified per patient (range, 1–11). The right side of Table 4 presents the probability of being loss-of-function intolerant (pLI) and combined annotation dependent depletion (CADD) scores. The pLI score reflects the tolerance of a target gene to the loss of its function on the basis of the number of protein truncating variants. A pLI score > 0.9 indicates that the mutation would cause disease with autosomal inheritance [17]. The CADD score is used to measure the deleteriousness of variants; the score predicts the pathogenicity of the variant [18]. Among the types of genomic functional effects, copy number alterations (CNAs) such as amplification and loss were recognized in 11 and 4 genes, respectively (Table 4).

**Table 4:** Summary of mutations

		Functional effect									
Gene	Total number	Nonsense	Missense	Frameshift	Non-frameshift	Splice	Allele fraction*	Amplification	Loss	pLI score	CADD score*
*PIK3CA*	9		3		6		0.3096 (0.0101–0.5875)			1	3.332(0.797-39)
*TP53*	7	1	3	2			0.5337 (0.0325–0.8020)			0.53	23(0.108-34)
*MYC*	4							4		0.99	
*PTEN*	3	1		1			0.8385, 0.5590		1	0.26	24.8, 2.877
*CDH1*	3									0.15	
*CDKN2A*	2								2	0.32	
*CDKN2B*	2								2	0.01	
*ZNF217*	2							2		1	
*MDM4*	2							2		1	
*IKBKE*	2		1				0.0572	1		unknown	3.158
*PIK3C2B*	2							2		1	
*MTAP*	2								2	0	
*FGFR1*	2							2		1	
*ZNF703*	2							2		0.36	
*NSD3*	2							2		1	
*SF3B1*	2		2				0.3314, 0.2628			1	5.916
*TBX3*	2			2			0.3940, 0.2493			0.98	5.758, 0.935
*FGF3*	2							2		0	
*FGF4*	2							2		0.14	
*FGF19*	2							2		0.57	
*SDHA*	2		1	1			0.4379, 0.5006			0	0.704, 24.9

*Median (range) was given when there were > 3 cases. pLI: probability of being loss-of-function intolerant; CADD: combined annotation-dependent depletion

## Discussion

Breast cancer is the second leading cause of cancer deaths among women recently. In Japan, 94,519 people were diagnosed with breast cancer in 2018. Regrettably, 14,779 (15.6%) patients died due to disease progression in 2020 [19]. If patients are affected by distant metastases, the chance of survival is decreased, as indicated in the 5-year relative survival rate with distant metastases of 39.3% [19]. It is a crucial obligation for clinicians specializing in breast cancer treatment to improve patient survival as much as possible. We have to devise a new therapeutic strategy, forsaking outdated considerations based on temporal changes in levels of tumor markers such as carcinoembryonic antigen and carbohydrate antigen 15 − 3. Since the phenotype of breast cancer is frequently altered at the time of metastasis, re-biopsy at metastatic sites and subsequent decision-making about appropriate therapy with consideration of subtype are recommended [20–22]. Modern advanced analytic techniques have opened up a new era for in-depth assessment of tumors using genomic assays. Currently, germline mutations in *BRCA1/2* and PD-L1 expression can be assessed easily with commercially available products [14, 23]. Obtaining this information has not been possible with classical IHC analyses. We clinicians have to take into consideration that genomic evaluation of cancer recurrence for personalized precision therapy is inevitable. As the latest frontier of comprehensive genomic profiling, we employed F1CDx as a NGS technology for the examination of entire exon regions of cancer-relevant genes. According to the literature research, Burstein MD et al. identified four distinct triple-negative breast cancer subtypes: (i) luminal androgen receptor, (ii) mesenchymal, (iii) basal-like immunosuppressed, and (iv) basal-like immune-activated by genomic profiling [24]. And the prevalence and distribution of immunotherapy responsiveness-associated gene mutations were identified by F1CDx [25].

The most prevalent altered gene was *PIK3CA*, with alterations detected in 9 (40.9%) patients. The median age of them was 63.1 years old (range, 43.7–78.2). *PIK3CA* alterations have been reported in 24–40% of patients with breast cancer [16, 26–30]. *PIK3CA*-activated alterations, like those in the 9 patients in the present series, might predict sensitivity to agents that target phosphoinositide 3-kinase, such as alpelisib. In the SOLAR-1 trial, median overall survival (OS) of the cohort with *PIK3CA* mutations (n = 341) was 39.3 months (95% CI, 34.1–44.9) for alpelisib in combination with fulvestrant and 31.4 months (95% CI, 26.8–41.3) for placebo-fulvestrant with a hazard ratio (HR) of 0.86 (95% CI, 0.64–1.15; P = 0.15). Alpelisib-fulvestrant failed to prolong OS, but median time to chemotherapy was significantly extended [3]. In the F1CDx report of each patient, this treatment was described as a clinically beneficial.

*TP53* alteration was the second most commonly altered gene in our study. The 7 patients (31.8%) with *TP53* mutations also had negative hormonal and HER2 status, i.e., triple-negative breast cancer (TNBC). And their median age was 46.4 years old (range, 33.4–81.9). *TP53* is one of the most frequently mutated genes in breast cancer, with mutations detected in 27–37% of breast carcinoma samples [31–36]. *TP53* mutations are also implicated in breast cancer susceptibility because *TP53* mutation carriers have an 18–60–fold increased risk for early-onset breast cancer [37–39]. And 3 patients with *TP53* alterations had high CADD scores and high allele fractions, above 0.5. However, no related pathogenic disorders were recognized in those 3 patients. Functional loss of the tumor suppressor p53, which is encoded by *TP53*, is commonly identified in aggressive advanced cancers [40]. Germline mutations in *TP53* are associated with autosomal dominant disorder Li-Fraumeni syndrome and early onset of many caners [41–43]. Unfortunately, there are no approved treatments to address *TP53* mutation or loss.

*MYC* amplification occurred in 4 patients: 3 patients with TNBC and 1 with luminal breast cancer. The median age of them was 47.4 years old (range, 38.0–66.8). The median amplification ratio was 2.165 (range, 1.9–2.52). *MYC* overexpression is reported to be higher in TNBC than in luminal breast cancer; it is associated with particularly poor outcomes and the loss of tumor suppressor pathways such as *p53* [40, 44, 45]. In our series, all 3 TNBC-patients with *MYC* amplification harbored *TP53* mutations with high allele fractions, ranging from 0.5337 to 0.6729.

*PTEN* alterations were detected in 3 patients (13.6%), 1 with loss and 2 with frameshift and nonsense mutations. The median age of them was 47.7 years old (range, 43.8–51.8). *PTEN* alterations are more frequently associated with triple-negative breast cancer than HER2 or hormone-positive breast cancer [46, 47]. Loss or reduction of *PTEN* expression is observed in 28% of invasive ductal carcinomas. It is associated with poor prognosis, including a shorter disease-free survival of approximately 2 years [48, 49]. In our series, the pathological diagnosis of the primary lesion in all 3 patients was triple-negative type breast cancer. Their disease-free interval was short: 22 months, 11 months, and 6 months. Their clinical courses seemed very aggressive. On the other hand, *PTEN* mutations cause inherited disorders such as Cowden syndrome. The incidence of Cowden syndrome is approximately 1 in 200,000 but is generally underestimated due to the high variability of this disorder [50]. In our series, the allele fraction of the 2 patients with frameshift and nonsense mutations was high at 0.8385 and 0.5590, respectively. Germline testing should be taken into consideration even when no family history of malignant cancer has been recognized. We could not perform germline testing because the patients did not consent to genetic testing.

*CDH1* mutations were identified in 3 patients. The median age of them was 68.2 years old (range, 48.7–82.1). *CDH1* encodes the transmembrane protein E-cadherin, which plays an important role in epithelial cell-cell adhesion [51]. Inactivation of *CDH1* is considered to be a genetic hallmark of invasive lobular breast carcinoma, with *CDH1* mutations in 46–65% of cases [50–53]. In our series, 2 patients with *CDH1* mutations were classified as having invasive lobular carcinoma and 1 was classified as having invasive ductal carcinoma (n = 1).

Another noteworthy mutation was detected in the *SDHA* gene. Mutations in this gene are associated with hereditary paraganglioma-pheochromocytoma syndrome and mitochondrial complex II deficiency (Clin Var, https://www.ncbi.nlm.nih.gov/clinvar/) [54]. In our series, this mutation was detected in 2 patients (54.9 and 65.4 years) with an allele frequency of 0.5006 and 0.4739, respectively. As their allele frequencies were relatively high, germline testing of *SDHA* should have been performed. However, we did not propose such testing because there was no confirmed family history.

In Table 5, we reevaluated the comprehensive genomic profile from the viewpoint of intrinsic molecular subtypes of breast cancer. In this series, there were 8 patients with TNBC (HR(-), HER2(-)) and 14 patients with luminal breast cancer (HR(+), HER2(-)). No significant differences were identified in the number of variants, including pathogenic variants and variants of unknown significance. However, TNBC had a significantly higher TMB score than luminal breast cancer. The elevation pf PD-L1 status was recognized in 4 (50%) patients with TNBC. It is reported that *BRAF* and *PBRM1* mutations would benefit by immune check point inhibitor, however, such genomic alterations could not be identified in those 4 patients with elevated PD-L1 status [25]. On the other hand, somatic mutations were expected to be induced with cancer chemotherapy. The lines of chemotherapy before F1CDx examination were considered to be low. Linear regression did not identify any correlations between the line of chemotherapy and the number of variants. The multiple correlation coefficient of those two factors was almost zero (P < 0.001).

**Table 5:** Biomarker and genomic findings by hormone receptor and HER2 status

Hormone and HER2 status*	Pathogenic variant	Number of patients	Line of chemotherapy^†^	Number of pathogenic variants^†^	Number of VUS^†^	TMB score^†^ (muts/Mb)
HR(-), HER2(-)	*TP53*	7	2 (0–3)	5.5 (2–9)	9 (2–20)	5 (5–11)
	*MYC*	3				
	*PIK3CA*	3				
	*PTEN*	3				
HR(+), HER2(-)	*PIK3CA*	6	1 (0–4)	4 (1–11)	6.5 (3–13)	3 (0–10)
	*CDH1*	3				
P value			0.2124	0.703	0.336	0.030

* ER(-) plus PgR(-) was described as HR(-). ER(+), regardless of PgR status, was described as HR(+)

^†^Medians (range). The HR(-), HER2(-) group had 8 patients. The HR(+), HER2(-) group had 14 patients

VUS: variants of unknown significance. TMB: tumor mutational burden

Herein we describe the case of a 67-year-old female who presented with a *BRCA2* reversion mutation. The patient had metastatic recurrence in a left subclavian lymph node. Because BRACAnalysis CDx® revealed a germline *BRCA2* mutation (c.6446_6450delTTAAA), olaparib was administered. This treatment remained efficacious for approximately 13 months. Subsequently, the lymph node showed re-growth, overcoming olaparib treatment. F1CDx performed using the re-biopsy specimen of the swollen left subclavian lymph node showed putative somatic *BRCA2* reversion mutations c.6419_6457del129 and c.6466_6469delTCTC with allele fractions of 0.0541 and 0.2772, respectively. The allele fraction of the germline *BRCA2* mutation diagnosed with F1CDx was 0.8739 in a re-biopsied lymph node. The secondary *BRCA2* mutations presented here removed an initial deleterious mutation and resulted in partial restoration of *BRCA* function [55, 56]. Therefore, abemaciclib, a cyclin-dependent kinases 4 and 6 (CDK4/6) inhibitor, and letrozole were chosen as the next therapeutic agents. The curative effect of this treatment was acceptable. The lymph node remained stable in size without re-growth for a year and half. The patient was classified as having stable disease overall.

One useful feature of the F1CDx assay is prediction of response to gene-targeted therapies by profiling the total number of synonymous and non-synonymous mutations across the coding regions of 324 cancer-associated genes. In our series of F1CDx assays, we could not propose an approved therapy available in Japan that is tailored to the genomic alterations for all patients. *PIK3CA* alteration, which is most prevalent mutation in our study, is eligible for alpelisib treatment, nevertheless the agent has not been approved in Japan. However, we identified several other alterations including *TP53* mutations and *MYC* amplifications, both of which are associated with poor prognosis in breast cancer. Those 2 variants were recognized in TNBC, which were considered to have aggressive features. We identified a *SDHA* mutation that was potentially a pathogenic germline mutation. TMB score, which was significantly higher in TNBC, was also evaluated with the F1CDx assay. TMB is reported to be associated with sensitivity to programmed death-1 and immune checkpoint inhibitors that target PD-L1 [6]. Throughout each patient’s long cancer journey, genomic alterations assessed with F1CDx might reflect transitory genetic variations affected by invasive treatment. To promote individualized care, clinicians should share personalized genetic information with patients and make evidence-based decisions in consideration of genetic heterogeneity.

For F1CDx, re-biopsy of the recurrent lesion is recommended. We experienced a case of a *BRCA2* reversion mutation and a case of HER2 receptor conversion. The F1CDx assay and FISH analysis of the resected lung metastasis revealed the loss of HER2 expression in a 62-year-old female patient. We should confirm whether biological features of tumors continue to change despite medical interventions.

This study had some limitations. First, although the gene assays of 2 patients, which consisted of assays performed after neoadjuvant chemotherapy and skin metastasis, respectively, were performed completely, the quality of the process was not optimal due to the low tumor nuclei content. Second, because the number of patients enrolled in this study was small, we evaluated only a limited range of genetic alterations. Third, the F1CDx assay can detect many CNAs, but CNA frequency in the study patients cannot be demonstrated with this assay.

In conclusion, the use of F1CDx in clinical settings will contribute to encouraging tailored precision treatment for patients with breast cancer. Clinicians should consider using comprehensive genomic profiling at turning points in the course of the disease.

## Data Availability

The data that support the findings of this study are available on request from the corresponding author. The data are not publicly available due to ethical restrictions.

## Abbreviations

F1CDx: FoundationOneCDx
F1CDx-Liquid: FoundationOne® Liquid CDx
ER: estrogen receptor
PgR: progesterone receptor
HER2: human epidermal growth factor 2
CEP 17: centromeric probe 17
PD-L: programmed death-ligand 1
IHC: immunohistochemistry
CPS: combined positive score
NGS: next-generation sequencing
TMB: tumor mutational burden
MSI: microsatellite instability
Mut/Mb: mutations per megabase
cfDNA: cell-free DNA
CTA: clinical trial assay
CI: confidence interval
FISH: fluorescence in situ hybridization
HR: hazard ratio
TNBC: triple-negative breast cancer
pLI: probability of being loss-of-function intolerant
CADD: combined annotation dependent depletion
CAN: copy number alteration
HR: hormonal receptor
CDK4/6: cyclin-dependent kinases 4 and 6
